# Barriers and facilitators to understanding of ADHD in primary care: a mixed-method systematic review

**DOI:** 10.1007/s00787-018-1256-3

**Published:** 2018-12-14

**Authors:** B. French, K. Sayal, D. Daley

**Affiliations:** 1 Division of Psychiatry & Applied Psychology, University of Nottingham, Nottingham, UK; 2 Centre for ADHD and Neurodevelopmental Disorders Across the Lifespan (CANDAL), Institute of Mental Health, University of Nottingham, Nottingham, UK

**Keywords:** ADHD, Systematic review, Primary care, Pathway to care

## Abstract

Attention deficit hyperactivity disorder (ADHD) is a developmental disorder affecting up to 5% of children and adults and is underdiagnosed in many European countries. The process of access to care for this disorder is complex and variable across countries. In general, those affected, or their caregiver, will seek help through their primary care practitioners who are then often responsible for referral to other professionals for diagnosis and provision of treatment. Previous studies have highlighted that many barriers to recognition exist in primary care settings (such as misconceptions, lack of education or lack of resources), preventing access to care for this population and potentially affecting diagnosis rate. This systematic review aims to establish the barriers and facilitators with regard to attitudes, beliefs and experiences of ADHD within primary care. Electronic searches of multiple databases identified 3898 articles of which 48 met our inclusion criteria—primary care professionals from any country, understanding, knowledge, awareness, attitude and recognition of ADHD. Four main themes were identified: (1) need for education, (2) misconceptions and stigma, (3) constraints with recognition, management and treatment, and (4) multidisciplinary approach. The findings suggest many interacting factors are at play in the recognition of ADHD by primary care practitioners with a strong recurring theme of a significant need for better education on ADHD. Implications for research and practice are discussed, suggesting that educational interventions for primary care practitioners could improve the recognition of ADHD in this setting.

**Electronic supplementary material:**

The online version of this article (10.1007/s00787-018-1256-3) contains supplementary material, which is available to authorized users.

## Introduction

Attention deficit hyperactivity disorder (ADHD) is a neurodevelopmental disorder affecting approximately 4% of children [1] leading to considerable functional impairment [2, 3] and often continuing on into adulthood. While evidence-based treatments can help manage ADHD, studies have shown that children with ADHD are underdiagnosed [4]. According to The National Institute for Health and Care Excellence (NICE) guidelines, 3–5% of children and 2% of adults in the UK population should be eligible for an ADHD diagnosis; however, in 2010, a diagnostic prevalence of only 0.506% was estimated for children and 0.016% in adults [5]. A greater understanding of the reasons behind these discrepancies is urgently required [6].

Although many factors influence service utilisation, such as parents and teachers’ perceptions, willingness to engage in help-seeking, or comorbid disorders [7–9], the first port of call in many countries is primary care and usually General Practitioners (GPs) who act as gatekeepers to care in the UK, for example. To receive an ADHD assessment and diagnosis if appropriate, children are referred to a psychiatrist or paediatrician through their GPs [10]; once a diagnosis has been made, GPs are then often involved in supporting the further management of children with ADHD and in liaising with parents and specialists. Their understanding of the condition is, therefore, crucial [11].

While there is no cure for ADHD, it can be managed with medication and non-pharmacological interventions [12]. However, unmanaged ADHD results in long-term impairments in many cognitive and behavioural domains [13]. Gaining timely access to care is, therefore, of great importance and research has demonstrated that this can be influenced by the knowledge and attitudes of health professionals [3], with limited GP recognition being a key barrier [8, 14, 15]. Most GPs will have undergone very limited training on ADHD, if any. In many countries, very few GPs have received official training [16]. Many GPs are not confident in recognising and managing ADHD, with lack of education about the disorder being a key component of their lack of confidence [17]. This limited recognition and education could be due to the lack of accurate knowledge and understanding of the disorder, scepticism and misconceptions, [18, 19] and many stigmas still associated with ADHD [12, 20].

A specific definition of what this review considers as primary care is given below but to facilitate the narrative of this review, due to the varied terminologies used across different countries, all terms referring to primary care personnel considered in this review such as GPs, family practitioners, and doctors will be described as primary care professionals (PCPs).

Some studies have looked at the attitudes of PCPs in relation to ADHD and two systematic literature reviews have summarised this evidence [6, 21]. The first review [21] looked at attitudes and knowledge of ADHD since 1994 when the Diagnostic and Statistical Manual of Mental Disorders 4th edition (DSM IV) was published, focused only on General Practitioners and only included studies about children. By not including all professionals in primary care and focussing on GPs only, this review missed studies published in the US which does not use the term GPs to refer to primary care professionals. This might have influenced the results as a considerable proportion of ADHD studies are from US research groups (i.e. half of the studies in our review). This review also excluded adults which is important as underdiagnosis of ADHD is even more prominent in adults [22] with stronger stigma and misconceptions as many health professionals still believe ADHD to be a childhood-only disorder [20]. The second review [6] looked broadly at the barriers and facilitators in the pathway to care for ADHD. While PCPs’ attitudes were part of the themes developed from the review, broader determinants were established such as parental involvement or issues with treatment. This review did not focus solely on PCPs’ understanding, the impact related to primary care being a small component of the review.

### Goals of the current review

The present systematic review aims to build on these two reviews [6, 22] by enhancing the focus on primary care through amending the selection criteria to include all primary health care settings in all countries, adult ADHD studies, all studies from inception of the databases and establishing facilitators and barriers to access to care for ADHD within the context of primary care. It sought to develop a segregated synthesis [23] of quantitative and qualitative research in an attempt to identify and synthesise current barriers and facilitators to the understanding of ADHD in primary healthcare, ultimately leading to improved recognition of ADHD.

## Methods

This review was written in accordance with the Preferred Reporting Items for Systematic Reviews and Meta-analysis Protocols (PRISMA-P) guidelines [24]. A protocol for the review was registered with the International Prospective Register of Systematic Reviews (PROSPERO; CRD42017071426) in July 2017.

### Inclusion criteria

#### Type of studies

Published and peer-reviewed quantitative and qualitative studies were included. The qualitative component of this review considered qualitative studies of any design exploring ADHD in primary care, including beliefs, understanding, attitudes, and experiences.

The quantitative component of this review included quantitative studies of experimental and observational designs (including, but not limited to cohort studies, case–control studies, randomised controlled trials).

Mixed methods studies were also included, and relevant qualitative and quantitative components were extracted separately.

#### Type of population

This review covered studies in primary care. Primary care is defined as the day-to-day health care provided in the community for people making an initial approach to clinics for advice or treatment [25]. Within the context of this review, primary care includes all public services health professionals that act as a first port of call for families and patients seeking medical advice (referred to as PCPs in this review). Therefore, professions such as physicians, family doctors, GPs, paediatricians, nurses and practitioners were considered depending on the country in which the study was conducted. Each study was thoroughly examined to determine, depending on the country of origin, whether the professionals studied were the initial approach healthcare providers. For example, in the UK, PCPs are often referred to as general practitioners but in the US they might be referred as paediatricians or family practitioners or physicians. However, US paediatricians can have primary and secondary care roles; therefore, careful consideration was given to the role when the term paediatrician was used in US-based studies. Studies involving private practices were excluded from countries where a public health system was available.

If more than one professional population was studied, primary care findings were extracted and reported separately if the study reported different professional groups separately. Studies from countries where PCPs are not gatekeepers and part of the primary care system were excluded if no reference to primary care settings was given.

#### Type of phenomenon of interest

This review examined the understanding of ADHD in primary care and looked at beliefs, attitude and knowledge, focusing on barriers and facilitators within these contexts. For the purpose of this study, barriers and facilitators were defined as perceived factors that hinder or facilitate the recognition or management of ADHD. As these definitions and concepts varied between studies, this review looked at these concepts broadly in the context of wider aspects of ADHD. This review considered studies focusing on understanding of ADHD throughout the lifespan and, therefore, included adult, adolescent and child studies.

#### Context

This review included any primary care settings. It took an international perspective and was not restricted to the English language, including relevant studies of all languages, translation being produced on an ad hoc basis. The time period of the review was not restricted and the search strategy covered all publications from database inception up to the 29th of January 2018.

### Exclusion criteria

Unpublished studies, literature reviews, case studies, opinion pieces, grey literature and non-peer-reviewed studies were excluded. Studies were also excluded if they did not specify the type of health professionals examined or did not report PCPs’ results separately from other groups. Studies focusing solely on ADHD medication and treatment effectiveness or evaluation were also excluded.

### Search strategy

Databases (PsychInfo, Embase, Scopus, ASSIA, Medline and Google scholar) were searched from inception to extract published studies. Following the search of the five main databases and removal of duplicates, an initial search and preliminary analysis were conducted of the subject headings (MeSH) and text words related to ADHD contained in the title and abstract (Supplementary Table S1). PROSPERO was also checked for ongoing or already published systematic reviews on the subject.

The search strategy comprised a combination of key words (e.g. ‘ADHD’, ‘Primary care’) and controlled vocabulary (e.g. ‘doctors’, ‘general practitioners’). The search was first performed on the 1st of May 2017 and updated on the 29th of January 2018. Date and language limits were not imposed.

While hand searching was not a major component of our planned search strategy, the reference lists of all selected papers that met the inclusion criteria were hand searched to check for additional studies.

### Study selection

Following the search, all identified citations were uploaded into Endnote and duplicates were removed. Two authors (BF and DD) screened the titles and then abstracts against the search inclusion criteria with 100% agreement. Full reports were obtained for all titles that appeared to meet inclusion criteria and imported into a dedicated folder on Endnote.

**Fig. 1:**
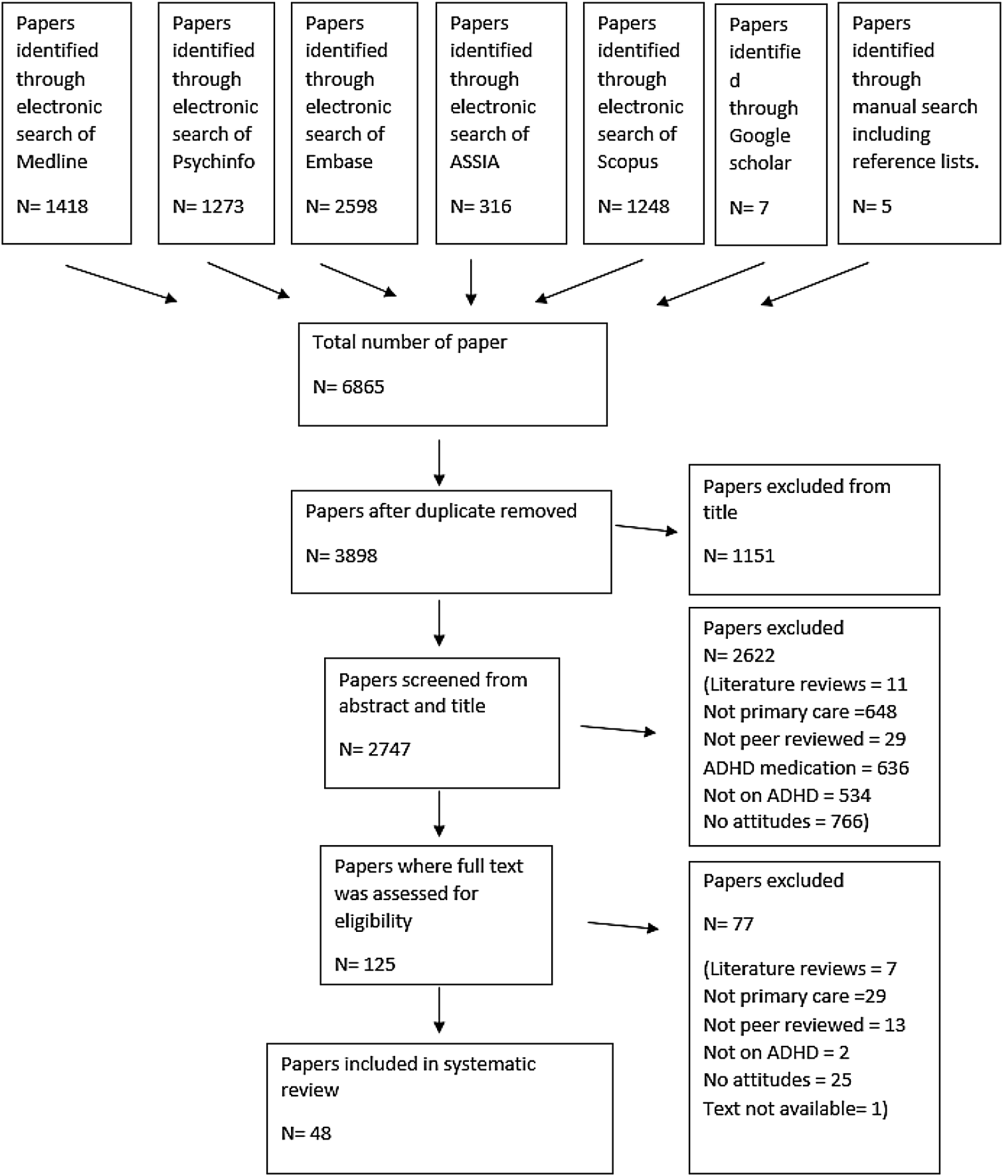
Flow diagram of the different selection processes

The same two review authors screened and assessed the full text in detail against the inclusion criteria. Disagreement on selected studies was resolved through discussion without the need to seek guidance from a third reviewer (KS). Studies that did not meet the inclusion criteria were excluded and are presented in the flow diagram below (Fig. 1), one full-text article was not available despite multiple requests from inter-library loan.

### Data extraction and outcomes

#### Data extraction

Two reviewers (BF and DD) extracted qualitative and quantitative data from the 48 included studies informed by a standardised data extraction tool for qualitative studies (JBI-QARI, [26]) and for quantitative studies (JBI-MAStARI, [26]), aiming to answer the review’s primary objectives. In the instance of studies reported in a foreign language, French studies were translated by the lead reviewer, a native French speaker, and translation was sought for other languages. Primary authors of relevant studies were contacted when additional information was needed.

#### Outcomes

The main outcome is the description and interpretation of PCPs’ understanding of ADHD including what hinders and facilitates their recognition of the condition. Multiple factors reported in the selected studies were evaluated such as beliefs, attitudes, knowledge and understanding. These factors were grouped into themes within the synthesis phase and are discussed in the context of barriers and facilitators.

### Assessment of methodological quality

Following mixed-method review guidelines [27], the process of quality assessment was separated between qualitative and quantitative studies. Two authors (BF and DD) critically appraised all selected studies for methodological quality using standardised quality appraisal tools for qualitative studies and quantitative studies [28]. These instruments assessed the quality of evidence across studies, including but not limited to criteria such as sampling strategy, analysis and sample size. Any disagreement between reviewers was resolved through discussion.

### Data synthesis

Due to the mixed-method nature of this review, a segregated synthesis was conducted where two distinct analyses involving qualitative and quantitative evidence were made prior to conducting a mixed-method synthesis [27].

A meta-synthesis summarised the qualitative findings, informed by JBI-QARI [26]. This aggregation or synthesis of findings generated a set of statements representing the aggregation, through assembling the findings rated according to their quality, and categorising them on the basis of similarity in meaning. These categories were then subjected to a thematic analysis informed by Braun and Clarke [29] to produce a single comprehensive set of synthesised findings that can be used as a basis for evidence-based practice. Where textual pooling was not possible the findings were presented in narrative form.

Quantitative data were synthesised in a comparable manner as statistical pooling was not possible due to high levels of heterogeneity within the included studies. The findings are presented in narrative form including tables.

The two analyses were aggregated by means of configuration [27]. The results of the syntheses were combined in the form of qualitative themes. The synthesised findings of the qualitative syntheses served as themes and together with the quantitative synthesis were summarised in thematic statements by the reviewers, involving the configurative conversion of all numerical results into qualitative thematic statements. These ‘converted’ findings and the qualitative thematic statements were then assembled. The aggregation/configuration of all themes generated a set of statements that represent the final aggregation, qualitative and quantitative findings complementing each other.

Two reviewers (BF and DD) conducted the syntheses in a sequential order, one reviewer developing the synthesis and the second checking the findings. Any disagreements were discussed and/or mediated by a third reviewer (KS).

The barriers and facilitators extracted for this review were categorised into four themes:

- Need for education—issues discussing the lack of training on ADHD for PCPs, lack of accurate awareness and a lack of confidence around ADHD.
- Misconceptions and stigmas—issues linking ADHD to general stigmatisation and misconceptions and the role of labels and media.
- Constraints with recognition, management and treatment—issues with time constraints and complexity of ADHD as well as issues with treatment options for ADHD.
- Multidisciplinary approach—issues with the role of different specialists, the role of the school, the parents and people with ADHD themselves.

## Results

### Study selection

The study selection process is shown in Fig. 1. Reasons for excluding trials after full-text assessment are provided in Supplementary Table S2. In total, 48 studies published between 1987 and 2017 met the inclusion criteria, of which 6 were qualitative, 2 mixed methods and 40 quantitative. The quantitative studies were all based on surveys and questionnaires with the exception of one free-listing exercise; while the qualitative studies were based on interviews (*n* = 4), focus groups (*n* = 2), no observational studies were identified. Characteristics of each study and their review themes are given in Table 1.

**Table 1:** Included study characteristics

Reference	Measure	Population of interest and sample size	Quality rating	Synopsis of findings	Themes
Qualitative studies					
Fiks et al. (2011b) US	Semi-structured interviews	30 paediatricians and 60 parents	High	Shared decision-making (SDM) GPs think SDM is more about convincing parents to follow their lead. Difficulty in determining how much involvement families should have Clinicians reported importance of involvement of other stakeholders, psychiatrists, schools in decision-making Half mentioned difficulty communicating with other specialists	Constraints with recognition, management and treatment Multidisciplinary approach
Guevara et al. (2005) US	Focus groups	3–4 focus groups of 19 family physicians	High	Highlighted breakdown of communication between parents, schools, physicians, not from a lack of will or desire. “System failure”—lack of accountability, discontinuity of care, lack of support, limited knowledge and resources and finger pointing Issues with treatment options available Limitation in training provided, even with previous knowledge, finding the constant change difficult to keep up with Lack of support from administration and lack of time to communicate with other schools	Need for education Constraints with recognition, management and treatment Multidisciplinary approach
Hassink-Franke et al. (2016) Netherlands	Interviews	15 GPs	High	Most GPs did not see a role for them in the diagnosis process Barriers: lack of knowledge and experience Too little time to get all information Resistance towards prescribing medication Importance of long-lasting relationships Felt more confident and competent after an online course on ADHD medication	Need for education Constraints with recognition, management and treatment Multidisciplinary approach
Klasen and Goodman (2000) UK	Semi-structured interviews on hyperactivity (not ADHD per se)	10 GPs and 37 parents	Moderate	Parents felt that GPs did not believe hyperactivity as a medical problem, most were unsure about boundaries between normality and abnormality Parents felt that professionals were against labels, that GPs were often badly informed and that it was a matter of chance whether they received useful help and information GPs felt that labelling did more harm than good Many GPs felt that parents’ views of hyperactivity as a medical problem were an attempt to avoid dealing with shortcomings in their parenting and an effect of dysfunctional families GPs were not aware of specialist help available in their area and not certain of whom to refer to Parents and GPs felt that information on hyperactivity was often conflicting and ambiguous GPs also felt they had not had sufficient training in assessment and treatment of hyperactivity	Need for education Misconceptions and stigmas Constraints with recognition, management and treatment Multidisciplinary approach
Klasen (2000) UK	Semi-structured interviews on hyperactivity	10 GPs and 37 parents	Moderate	Only 2 out of 10 GPs had diagnosed children with hyperactivity 2 felt labelling ADHD was not useful Uneasiness around medication	Need for education Misconceptions and stigmas Constraints with recognition, management and treatment
Shaw et al. (2003) Australia	Focus groups	28 GPs in 4 groups	High	GPs believed the main causal factor of ADHD was increased stress in daily life, contributing to difficulties in parenting. The use of labels has led to labelling bad parenting as ADHD Importance of involvement of specialists Time, training needs and medication management identified as constraints in ADHD management Lack of knowledge and training, need for more multidisciplinary support Negative media representation of medication	Need for education Misconceptions and stigmas Constraints with recognition, management and treatment Multidisciplinary approach
Mixed methods studies					
Leslie, et al. (2006) US	Vanderbilt rating scale. Likert scale interviews	16 paediatricians	Moderate	Need for better tools and training to identify discrepancies between parents and teachers’ reports Material accessible for families from different background and in different languages	Constraints with recognition, management and treatment
Salt et al. (2005) UK	Questionnaire survey and interviews	GPs. 93 surveys and 13 interviews	High	Mixed results on factors believed to influence ADHD, causes and diagnosis procedures Some thought quality of parenting was relevant 75% thought some non-ADHD symptoms were ADHD symptoms, despite non-inclusion in DSM criteria Only 3 GPs in surveys restricted themselves to the three main symptoms GPs agreed of the strong stigmatisation and controversial nature of ADHD; importance of the media in attitude towards ADHD All GPs were uncertain about prevalence rates in UK Lack of adequate training on ADHD	Need for education Misconceptions and stigmas Multidisciplinary approach
Quantitative studies					
Alder et al. (2009) US	Survey on adult ADHD. Likert scale	400 primary care physicians	High	Only 13% reported that they had received good training 77% believe adult ADHD is not well understood 72% agree that it is more difficult to diagnose in adulthood than in childhood 48% reported lack of confidence in diagnosing adult ADHD and 44% believe that there are no clear criteria 75% reported poor quality of assessment tools with 85% indicating they would take a more active role if a reliable tool existed	Need for education Constraints with recognition, management and treatment
Ayyash et al. (2013) UK	Delphi methodology from consensus statement to questionnaire. Level of agreement on a scale of 1–4 of 40 statements	122 specialist of which 6 trainee doctors	Moderate	Variation in scoring on ADHD consensus between subgroups, trainee doctors had the lowest agreement scores The variation in scoring across each of the subgroups of respondents may prove useful in understanding the different perspectives offered by each sub-group Shared cared, integrated pathways between primary and secondary care Need to raise awareness in primary care regarding ADHD, especially with GPs. Commissioning may be developed collaboratively across multiple GP consortia. Failure to treat ADHD effectively has significant social and economic impacts Primary care clinicians need to be educated to recognise the diagnostic signs of ADHD	Need for education Multidisciplinary approach
Ball (2001) UK	Questionnaire on attitudes and use of methylphenidate	150 GPs	Moderate	Only 6% had received formal ADHD training 28% gained information from articles and 21% from the media 11% do not prescribe ADHD medication due to lack of knowledge Complex views on the role of different professionals Over 60% felt they would change their view with clearer advice from specialists and clear protocol on monitoring 80% wanted further training and 88% specifically on medication	Need for education Constraints with recognition, management and treatment
Baverstock et al. (2003) UK	Questionnaire with 11 open-ended questions	45 GPs in university and college settings.	Low	Transitional care for university students 39 GPs had not attended any courses on ADHD GPs commented that it is likely to be an underestimate (due to complexity and inaccuracy in the way ADHD is recorded) and that most students with a diagnosis are from the US. Some surgeries said that they had no awareness of university students with ADHD, unless student were on medication Patient fail to attend follow-up	Need for education Multidisciplinary approach
Chan et al. (2005) US	Survey with 53 Likert scale questions	861 paediatricians and family physicians	High	Variation in time and number of visit to gain evaluation, getting teacher information is difficult Only 57% use formal criteria to make a diagnosis, of which only 27% used DSM. Most do not follow AAP guidelines Increased volume of ADHD evaluation associated with increased use of formal criteria and increased used of teacher/school information Decreased volume of ADHD evaluation associated with increased likelihood of using laboratory test (lead level, thyroid) and more likely to feel inadequately trained 36% felt inadequately trained and 66% inadequately trained with comorbid	Need for education Constraints with recognition, management and treatment Multidisciplinary approach
Clements et al. (2008) US	Survey with Likert scale	35 paediatricians and family physicians with ADHD patients	Moderate	80% used parent and teacher information for diagnosis 74% reported getting information on ADHD through self-training, 80% on continuing medical education and 45% from medical school	Need for education
Copeland et al. (1987) US	Survey, 21 multiple-choice questions	290 paediatricians	High	Only 20% based their definition of ADHD on DSM Majority identified main symptoms, 35% said social difficulties and anger where also symptoms 79% said increased activity in GP office contributed to diagnosis and 20% dysmorphic features Over 60% used parents and teachers scales	Need for education
Daly et al.(2006) US	Survey of 18 questions	303 family physicians	High	54% were not aware of AAP guidelines 90% used DSM diagnostic criteria 77% used lab test (lead, EEG, etc.) Barriers to diagnosis included, lack of training and education, time constraints and complexity	Need for education Constraints with recognition, management and treatment
Dryer et al. (2006) Australia	117 items questionnaire, Likert scale	670 medical professionals of which 82 GPs	High	GPs thought that behaviour and concentration were characteristics of ADHD as well as low self-esteem and adjustment problems For causal factors, GPs agreed that it was mainly due to brain function as opposed to home, school or toxins	Need for education
Evink et al. (2008) US	Questionnaire and vignettes	66 physicians	High	Comparison between different types of physicians 55% of family physicians vs 100% of paediatricians use DSM criteria 100% will seek specialist input when presented with complicated cases Main difference in treatment and assessment is in medical specialty Pressure from parents and schools	Multidisciplinary approach
Fiks et al. (2011a) US	Free listing and interviews of word related to ADHD	30 paediatricians and 60 parents	High	ADHD was linked to the words school, impulsive, hyperactive and focus Clinicians associated help with medication, time (negative), side effect, psychologist and frustration Talking to families was associated with time, learning and explaining	Need for education Constraints with recognition, management and treatment
Fuermaier et al. (2012) Netherlands	Stigma questionnaire on adult ADHD	228 professionals of which 74 physicians	High	Shows that a control group (matched in age, sex and education) and physicians do not differ in level of stigmatisation towards ADHD The only subscale where they showed lower stigmatisation is misuse of medication Reflect different training and experience and different dimensions of stigmatisation	Need for education Misconceptions and stigmas
Gamma et al. (2017) Switzerland	Survey on ADHD	75 physicians	Moderate	44% of presenting cases were diagnosed by PCPs Difference in diagnosis and management between GP and paediatricians Only 7% of PCPs felt competent in diagnosing ADHD, lack of competence the primary reason for not diagnosis GPs felt less competent than paediatricians	Need for education
Gardner et al. (2002) US	Survey on mental health with small elements of ADHD	395 primary care clinicians	High	Physicians were more likely to find ADHD in boys when presented with boys and girls with similar levels of parent reported problems Therefore, bias of treatment for different genders	Misconceptions and stigmas
Ghanizadeh and Zarei (2010) Iran	Self-reported questionnaire to assess knowledge and attitude	665 GPs	Moderate	20% reported ADHD is not a serious problem, 1/3 believed sugar is a cause Nearly all reported higher risk of delinquency, 80% believe it is a risk factor for truancy Different beliefs on IQ and educational levels Half believed it is due to dysfunctional families, only 6% believed it can be lifelong Not sufficient information about ADHD	Need for education Misconceptions and stigmas
Gomes et al. (2007) Brazil	Interviews	2117 professionals of which 128 general practitioners	High	7% of GPs did not know of ADHD even after reading a definition GPs expressed the least agreement with the statement “ADHD must be treated with medical products” 5% believed it is not a disease 19% believed you can leave without treatment	Need for education Misconceptions and stigmas Constraints with recognition, management and treatment
Goodman et al. (2012) US	Survey with clinical vignette on adult ADHD	1924 professionals of which 1216 primary care physicians	High	30% reported being not confident in diagnosis, 38% in treatment, 35% in managing adult ADHD Greatest barrier was limited experience Reported difficulty distinguishing ADHD from other things Main barrier: complexity of disorder, stigma, concerns around meds and adherence to therapy Gap in communication between specialists Almost 50% believed ADHD is caused by absent parent or bad parenting	Need for education Misconceptions and stigmas Constraints with recognition, management and treatment Multidisciplinary approach
Heikkinen et al. (2002) Finland	Questionnaire, 16 items, not just about ADHD	499 physicians	High	44% of male and 60% of female physician felt confident in their skills in assessing ADHD	Need for education
Jawaid et al. (2009) Pakistan	Questionnaire	194 primary paediatric care providers	High	Colleagues were reported as the main source of information Only 13% of GPs and 21% of paediatricians were shown to have sufficient knowledge 50% showed inadequate knowledge No training for GPs in ADHD in Pakistan	Need for education
Kwasman et al. (1995) US	A 48-item survey Likert scale	380 paediatricians	High	8% reported being “burned out” by ADHD children 39% reported barriers in time required Want more interdisciplinary contact, Only 8% follow-up Misconceptions about ADHD including poor dieting, child does it on purpose, medication can cure ADHD and children outgrow ADHD 44% believe ADHD medication is addictive	Misconceptions and stigmas Constraints with recognition, management and treatment Multidisciplinary approach
Kwasman et al. (2004) US	51-Item survey	786 school nurses	High	89% attended presentation on ADHD Most agree that they tried to get written report from school to physician Most disagree of integration of communication between school and physician Most disagreed that physicians did a good job at educating parents and children about ADHD Higher estimate of boys vs girls	Need for education Misconceptions and stigmas Multidisciplinary approach
Lanham (2006) US	55-Item survey	235 physicians	High	Only 22% are familiar with guidelines 70% use child behaviour in office to make an official diagnosis	Constraints with recognition, management and treatment
Lian et al. (2003) Singapore	Cohort study on developmental disorders—4 questions on ADHD	48 GPs	Moderate	31% agreed that children may show all signs at home but not in school 25% believed sugar to be the cause 73% agreed that it improved in adolescence 85% believe that medication alone is sufficient	Need for education Misconceptions and stigmas
Louw et al. (2009) South Africa	Surveys 22-item multiple-choice questions	229 GPs	High	57% reported average to good knowledge of ADHD in children. Only 10% in adults 7% felt they had adequate training in children and 1% in adult Self-study most prominent education tool, lack of training at university level Most felt the need to know more about ADHD, in adults 89% and children 81% Need for appropriate screening tools Main barriers in management are uninformed parents, limited funds, time and difficult parents	Need for education Constraints with recognition, management and treatment Multidisciplinary approach
Miller et al. (2005) Canada	Questionnaires	405 GPs and FPs	High	47% reported low comfort with diagnosis, 52% high 51% low skill in diagnosis, 48% high 51% low comfort with management, 48% high 50% low effectiveness in management, 49% high Comfort skills are a predictor of GPs’ tendency to take responsibility and are related to previous educational exposure	Need for education
Morley (2010) US	Case studies vignettes with a survey	187 primary care physicians	High	Race and insurance status do not have an effect on diagnosis Respondent effective at discriminating between ADHD cases or not	
Murray et al. (2006) UK	Questionnaires	40 GPs	Low	Only 22% were aware of the three diagnosis criteria Almost half identified the need for more information 7 thought causes of ADHD were due to family management approaches	Need for education Misconceptions and stigmas
Power et al. (2008) US	Questionnaire of 24 items with Likert scale	121 primary care providers	High	PCP believe assessing ADHD is within their scope of practice as well as prescribing medication Issues with initiating communication with school professionals Additional training related to assessment, school collaboration, family education and collaboration with mental health providers	Need for education Multidisciplinary approach
Quiviger and Caci (2014) France	Questionnaire of 23 items	57 paediatricians	High	13 out of 49 did not know what TDAH (Trouble Déficit de l’Attention/Hyperactivité—ADHD) stood for 72% responded having insufficient training on ADHD Education on ADHD is mainly self-taught from articles, colleague or internet 24% thought it was a disorder constructed abroad and imported to France, 36% thought it was societal, 15% believed it is due to bad parenting 77% believed mothers worry too much about hyperactivity 62% based their decision on the child’s behaviour in the practice	Need for education Misconceptions and stigmas Constraints with recognition, management and treatment
Ross et al. (2011) US	38-Item cross-sectional survey	100 primary care paediatricians	High	Communication with psychiatrist is low and changeable, would prefer closer collaboration 15% reported receiving communication with psychiatrist Depend on parents to provide information	Multidisciplinary approach
Rushton et al. (2004) US	37-Item survey about diagnosis and treatment measures	723 paediatricians and family physicians	High	77% familiar with AAP guidelines and incorporated them in their practice Laboratory test still conducted by up to 39% (lead, iron) 20% believe parents are reluctant to accept diagnosis 55% believe teachers pressurise them to get diagnosis and 70% to prescribe meds 43% believed of misuse of meds which was associated with less prescription Most did not believe stigma was a barrier to access to care Lack of awareness of guidelines, only 44% used DSM criteria	Need for education Constraints with recognition, management and treatment Multidisciplinary approach
Sayal et al. (2002) UK	Questionnaire	16 GPs	High	GP were less likely to agree that children could managed solely with primary care	Multidisciplinary approach
Shaw et al. (2002) Australia	Questionnaires	399 GPs	High	A majority believed inadequate parenting was influential Importance of multimodal assessment Variation in DSM knowledge of features of ADHD, lack of confidence 17% believed stimulant is an inappropriate treatment Most GPs were unhappy managing respondents in general practice as it is too difficult and time consuming	Need for education Misconceptions and stigmas Constraints with recognition, management and treatment Multidisciplinary approach
Stein et al. (2009) US	8-Page survey with fixed responses	745 paediatricians	High	12% reported they neither treat nor report ADHD 53% responded that paediatricians should not be responsible for referring ADHD Continuity of care associated with enquiring and treating ADHD Debate over whether prevalence in practice and higher level of attendance at lectures/conferences are causes or consequences of inquiring and treating/managing. Once paediatricians are more aware of a problem, it is likely that they will pay more attention to it	Need for education Multidisciplinary approach
Thomas et al. (2015) US	37-Item survey with closed responses	298 professionals of which 59 physicians and 138 nurses	High	Only 38% believed ADHD to be a problem Half of respondents felt comfortable in their ability to recognise ADHD symptoms, nurses least comfortable Over 85% stated the need for more research in college students and ADHD	Need for education Constraints with recognition, management and treatment Multidisciplinary approach
Venter et al. (2003) South Africa	51-Item survey	143 GPs	Moderate	Problems area identified were coordination of intervention and liaising with schools 45% found parents difficult Management of ADHD could be improved by teacher education, parent education, interdisciplinary contact and improved training of medical professional The majority believed chaotic home situation and bad parenting were strong influences 68 and 67% of GP and nurses thought it was difficult to diagnose ADHD in college student	Need for education Misconceptions and stigmas Multidisciplinary approach
Ward et al. (1999) Canada	One-day course. Three part needs assessment: 42-item survey	100 family physicians 34 provided data before and after	High	An educational program showed significant difference about ADHD knowledge pre- and post-tests, and altered management of ADHD Pre-course, 17% referred for diagnosis with a minimum of history taking, 4% post-course	Need for education
Wasserman et al. (1999) US, Puerto Rico and Canada	Questionnaire	401 paediatricians	High	AHP (attentional and hyperactivity problems) rather than ADHD DSM criteria used in only 38% and school report in only 53%. Lack of standardisation in primary care assessment Children 7–10 years old, twice as likely to be diagnosed to those older with higher scores No evidence of used of labels by clinicians to children with family or social issues, racial or ethnic status. Gender bias	Misconceptions and stigmas
Williams et al. (2004) US	Interviews on behavioural health diagnosis	47 paediatricians	High	High level of comfort in making ADHD diagnosis and prescribing meds 48% spend time focused on ADHD, information about cause of the disorder, school modification, organisation skills, parenting Great interest in future training for update on ADHD, not so much basic information	Need for education
Wolraich et al. (2010) US	Surveys in 1999 and in 2005	551 paediatricians in 2005, 452 in 1999	High	Increase in use of APA guidelines over the two surveys More used diagnostic criteria More used both teacher and parent rating scales Large proportion in both surveys felt training in treatment and evaluation was inadequate	Need for education

A range of countries were represented with most of the studies originating from the US (23 studies), UK (eight), Australia (3), Canada (4), Netherlands (2), South Africa (2) and 1 each from Iran, Brazil, Finland, France, Pakistan, Switzerland and Singapore.

### Data methodological quality

Results of study quality are reported in Table 1. Following Kmet, Lee and Cook’s guidelines [28], an original quality score from 0 to 1 was calculated for each study. Scores were then classified into low (0–0.44), moderate (0.45–0.69) and high (0.70–1.00). Study quality was assessed by two reviewers (BF and DD). Agreement between reviewers was 88% overall (92% for quantitative studies, 85% for qualitative studies).

The studies showed some variation in their quality. The average quality score was 0.73 with 36 studies receiving a high-quality rating, 10 a moderate rating and 2 a low rating, (quantitative studies mean score of 0.75; qualitative studies mean score of 0.70). The two studies that received a low-quality rating were not used to inform our review results and conclusions.

### Data extraction and summary of results

#### Need for education

The main theme highlighted by this review related to the need for education on ADHD. Thirty-seven papers identified issues related to a lack of education on ADHD, representing a wide range of countries, 14 from the US [30–43], 7 from the UK [3, 11, 16, 44–47], 3 from Australia [48–50], 2 from the Netherlands [51, 52], 2 from South Africa [53, 54], 2 from Canada [55, 56] and 1 each from France [57], Singapore [58], Pakistan [59], Finland [60], Brazil [61], Iran [62] and Switzerland [63]. These papers highlighted both a lack of initial training, as well as inadequate training on ADHD. They also highlighted a lack of awareness, experience, understanding and knowledge of ADHD. Most PCPs also reported a lack of confidence about ADHD and in treating ADHD. These factors often hindered general knowledge and understanding of ADHD.

- Lack of initial training and inadequate training

Lack of training on ADHD was recorded by many studies. This included studies reporting a lack of training especially at undergraduate and postgraduate medical education levels [3, 16, 30, 38, 45, 53, 57] with studies suggesting that 1% [53] to 28% [57] of PCPs received specific training about ADHD. In a quantitative study from the UK, Ball [16] found that only 6% of 150 PCPs surveyed received formal training on ADHD and 80% reported wanting further training. This was more prominent for adult ADHD with two studies reporting a greater lack of education [30, 53]. Even when studies reported training on ADHD, the training was often considered by PCPs to be inadequate [11, 31, 59] with up to two-third of PCPs feeling inadequately trained to evaluate children with ADHD [31]. While a strong need for training on ADHD in general was observed, three studies also highlighted the importance of updated training incorporating new knowledge [37, 39, 42]. This lack of education affected many aspects of the primary care experience of patients from referral and diagnosis [44, 59] to management [50] of ADHD.

- Lack of awareness, experience, understanding and knowledge of ADHD

The need for education was also highlighted through the lack of general awareness, experience, understanding and accurate knowledge of ADHD. While two studies directly reported a lack of knowledge and experience [36, 52] as a barrier to dealing with people with ADHD, eight investigated these concepts through knowledge of the DSM criteria or clinical guidelines [31, 33, 34, 47, 64–67]. Accurate knowledge of guidelines and procedures for identification of ADHD were low; for instance, only 20% [33] —27% [31] of PCPs were using DSM criteria and only 20% were using official guidelines [65]. One study from the UK [11], using a mixed-method approach, found that 75% of PCPs could not identify ADHD DSM criteria correctly and all PCPs were unsure of ADHD prevalence and diagnostic procedures. Two studies investigated these concepts through questions about several factors including treatments, prevalence and symptoms, reporting different levels of knowledge and awareness throughout, with inaccurate beliefs such as believing that there is no need for treatment [61] or that ADHD was not a medical problem [3]. Additionally, very few studies reported a majority of PCPs in their sample being able to accurately identify ADHD characteristics. Two studies reported that PCPs did not know what the acronym TDHA (ADHD in French) stood for [57] or that they did not know about ADHD even after reading its definition [61] demonstrating that a lack of general knowledge about ADHD was highly evident.

- Lack of confidence about ADHD and its treatment

The final aspect relating to the need for education highlighted PCPs’ lack of confidence about ADHD, most specifically in treating ADHD. This review and the included studies focus principally on the recognition of ADHD but a few studies also raised the issue of a lack of confidence, encompassing treatment and management. In these studies, it is unclear whether the lack of confidence is solely around recognition or all aspects of ADHD management; therefore, it was important to include this aspect in our findings. While two studies reported a general lack of confidence [30, 49], three [36, 55, 63] reported low confidence and competence in diagnosis and management of ADHD. Some studies reported a lack of confidence toward treatments, with PCPs reporting being uncomfortable with medication for ADHD. Goodman et al. [36] reported that 38% of PCPs had no confidence in treating ADHD, Alder et al. [30] also highlighted a considerable lack of confidence in treating adults with ADHD and Ball [16] reported that 11% of PCPs were not willing to prescribe medication at all due to lack of knowledge, while 88% of PCPs wanted further training in the drug treatment of ADHD.

##### Facilitators

While the need for education underpinned many barriers and issues towards the overall understanding and knowledge of ADHD, a few positive outcomes were also observed. In contrast to our overall findings where a lack of knowledge and confidence was evident, three studies reported above-average ratings of confidence and high knowledge of ADHD [41, 42, 60] and Evink et al. [64] reported that all paediatricians in their studies used DSM criteria. The reasons for these different findings are unclear but could be due to the high number of paediatricians in the studies who might have received specialist paediatric training.

Despite the overall lack of training and awareness of ADHD, studies reported that PCPs had a keen interest in wanting to gain more knowledge [42], with strategies such as asking colleagues, self-education, and online enquiries. [16, 40, 53, 57, 62]. Two studies [52, 56] explored the benefits of educational programs for PCPs and reported an increased awareness and confidence in ADHD after taking part; the first study [52] focused on an educational program for prescription and monitoring of ADHD medication, while Ward et al. [56] evaluated a 1-day course which aimed to teach PCPs to manage ADHD and observed a significant difference between pre-test and post-test knowledge. Furthermore, Wolraich et al. [43] reported a marked increase in the use of APA guidelines between 1999 and 2005 by PCPs (13%–50%), suggesting an increased interest and awareness in ADHD.

#### Misconceptions and stigmas

Linking to the previous theme, misconceptions and stigmatisation surrounding ADHD were often strongly present in the literature. This notion was explored by different studies, either directly reporting the experience of stigma within primary care or reporting inaccurate facts about ADHD; reflecting gender biases (‘it only happens in boys’), misleading causes of ADHD (due to high sugar level or lead poisoning) or most prominently, that ADHD is primarily caused by bad parenting. Seventeen studies discussed elements related to misconceptions, five from the US [36, 38, 67–69], four from the UK [3, 11, 46, 47], two from Australia [49, 50], and one each from the Netherlands [51], Iran [62], Brazil [61], Singapore [58], France [57] and South Africa [54].

- General stigmas and misconceptions

Most studies reported general misconceptions about ADHD. In a mixed-method study in the UK, Salt et al. [11] reported that over 50% of PCPs agreed on the controversial nature, the strong stigmatisation of ADHD and the disadvantages the diagnosis brought. In a quantitative survey of 380 US PCPs, Kwasman et al. [69] reported strong misconceptions about ADHD, including: ADHD is “caused by poor diet” (21% agreed), “the child does it on purpose” (15%), “medications can cure ADHD”(10%) and “ADHD medications are addictive” (48%). Many studies reported participant views that sugar levels were a cause of ADHD [58, 62, 69] while others reported a gender misconception that ADHD was only present in boys [38, 68]. Other misconceptions were more surprising with Ghanizadeh and Zarai [62]; for instance, reporting that 82% of PCPs believed children with ADHD misbehaved primarily because they do not want to obey rules and do their assignments, while Quiviger and Caci [57] stated that 24% of the PCPs surveyed thought it was a disorder constructed abroad and imported into France.

While it could be expected that PCPs should not hold stigma towards ADHD due to their expected knowledge of the disorder, in a quantitative study in the Netherlands on stigmatisation towards ADHD, Fuermaier et al. [51] reported no difference in stigmatisation levels between physicians and a control group of non-medical professionals.

- Bad parenting

Ten studies reported that PCPs believed ADHD was due to bad parenting [11, 36, 46, 47, 49, 50, 54, 62], numbers varying from 15% [57] to over half [36, 62] of PCPs surveyed believing that dysfunctional families were predominately to blame for ADHD symptom expression. In semi-structured interviews with UK PCPs and parents, Klasen and Goodman [3] reported that most GPs saw symptoms of hyperactivity (one of the three symptom clusters of ADHD) as an effect of dysfunctional families and many felt that parents’ views of hyperactivity as a medical problem were an attempt to avoid dealing with possible shortcomings in their parenting practices.

- The role of the media and labels

Four studies linked the presence of misconceptions with negative media coverage and the use of labels. Klasen and Goodman [3], for instance, reported that parents felt PCPs were against labels, trying to normalise hyperactive behaviours, while Klasen [46] reported that 25% of PCPs felt labelling was not useful. Salt et al. [11] highlighted, through a targeted questionnaire, the influence of the media in the general public’s conception of ADHD, whereas Shaw et al. [50] argued that negative media coverage and labels affect the representation of medication and had led to labelling bad parenting as ADHD.

##### Facilitators

Although very few facilitators can be observed within this theme, it is important to note that the concepts of misconception and stigma were only explored in a third of included studies suggesting stigma about ADHD did not emerge from studies as much as might have been anticipated. Studies identifying stigma reported stigma from a wide variety of different countries and cultures, suggesting that stigma surrounding ADHD is not specifically culturally determined.

#### Internal and resource constraints with recognition, management and treatment

As PCPs are often responsible for the recognition and management of ADHD, a few barriers were observed surrounding these procedures. The first considered the barriers experienced around recognition, referral and diagnosis of ADHD, mainly referring to resource constraints such as time and the need for appropriate screening tools. With regard to treatment options, the main barriers observed included the limited treatment options available and uneasiness around medications. Twenty studies discussed aspects related to recognition and treatment, with eleven studies from the US [30, 31, 34–37, 41, 66, 69–71], three from the UK [3, 44, 46], two from Australia [49, 50] and one each from Brazil [61], the Netherlands [52], France [57] and South Africa [53].

- Time constraint and complexity of ADHD

The resource constraint mainly experienced in the recognition and management of ADHD is in relation to time and the complexity of ADHD. Many studies found that the time necessary to gain all the relevant information was often too demanding [31, 34, 35, 49, 50, 52, 53, 69] especially taking into consideration the complex nature of ADHD [34, 36, 41]. After interviewing 19 PCPs in focus groups in the US, Guevara et al. [37] reported that limited resources and lack of time to communicate with schools led to limited access to care, whilst Klasen and Goodman [3] found in their interviews in the UK that information necessary for management and recognition is often conflicting and ambiguous. Five other studies mentioned the need for better assessment tools [3, 35, 54, 71], especially for adults [30]. Finally, one other barrier experienced in relation to time and resource constraints is that PCPs make decisions on assessment for referral based on the child’s behaviour in their office [33, 57, 65], which can lead to potential misdiagnosis as the child might behave very differently at home or school [72].

- Treatment

Considerable issues were also highlighted around treatment: the lack of available treatment options as well as uneasiness around medication. While studies reported a general uneasiness with ADHD medication [36, 38, 41, 50], this at times led to resistance or refusal to grant prescriptions by PCPs [16, 52, 66]. In a series of interviews with 128 PCPs, Gomes et al. [61] reported high levels of uneasiness around medication, limited knowledge of treatment options, and a lack of knowledge of the pros and cons of medication and other treatments. This reflected other findings describing confusion around treatment options in relation to professionals’ knowledge of what is available and limited availability of treatment [3, 36, 70].

##### Facilitators

Despite the constraints explored in these studies, attempts to address these issues were reported in only two studies. After participating in a 1-h educational online course on ADHD medication, Hassink-Franke et al. [52] found that most PCPs felt more confident and competent about prescribing and monitoring medication. As this was a qualitative study, information was not available on the degree of change of confidence in the participating PCPs. Ward et al. [56] evaluated a 1-day course which aimed to help PCPs to manage ADHD. Results demonstrated some impact on practice in the form of increased levels of ADHD referrals. However, the study was based on only 34 clinicians, was not controlled and did not verify the appropriateness of referrals.

#### Multidisciplinary approach: the role of other specialists, teachers, parents and patients

The final theme encompassed the concepts of a multidisciplinary approach. This mainly referred to the role of different specialists and the importance of shared care. However, it also included the role of other parties involved such as the people with ADHD, parents and teachers. Twenty-two studies explored issues pertaining to a multidisciplinary approach, twelve from the US [31, 36–41, 64, 66, 69, 70, 73], five from the UK [3, 43, 454, 12, 15], two from Australia [49, 50], two from South Africa [53, 54] and one from the Netherlands [52].

- The role of specialist and the importance of shared care

When discussing the concept of the multidisciplinary approach, many studies explored the communication between specialists, principally between primary and secondary care. With the belief that integrated care pathways and a collaborative approach is essential [15, 40, 44, 64], issues with communication between specialists was expressed as a major barrier [36, 39, 49, 50, 69]. In semi-structured interviews in the US [70], PCPs reported the importance of involvement of other stakeholders, psychiatrists, and schools in decision-making and over half of the professionals interviewed mentioned difficulties in communicating with other specialists. Furthermore, Ross [73] reported that only 15% of PCPs surveyed received communication from psychiatrists. Guevara [37] found similar issues with communication and a need for shared care; however, this paper acknowledged the breakdown of communication between parents, schools and physicians but not from a lack of will or desire, rather as a ‘System failure’—lack of accountability, discontinuity of care, lack of support, limited resources and finger pointing.

Ambiguity about the role of different professionals [11, 40, 41, 52] was also noted as a barrier to access to care. Klasen and Goodman [3] highlighted that most PCPs were not aware of specialist help available in their area and were not certain of whom to refer to.

- The role of the school, parents and patients

Communication with other parties such as schools, parents and people with ADHD themselves was also reported as being a barrier. Four studies, for instance, mentioned that patients failing to turn up for appointments limited the PCPs’ ability to assess them and provide the right care [36, 44, 45, 69]. Other studies found that PCPs experienced considerable difficulties in getting information from parents and schools [31, 39, 73] as well as reporting feeling continued pressure for diagnosis from schools and parents [53, 54, 64]. In a US survey of 723 PCPs, Rushton et al. [66] found that 55% felt a strong pressure from teachers to diagnose ADHD while 70% felt pressure to prescribe medication. Kwasman et al. [38] reported that their large sample of school nurses highlighted a lack of multidisciplinary communication between PCPs and school staff and suggested that PCPs and schools would benefit from a greater understanding of the contributing that each could make to an effective ADHD assessment.

##### Facilitators

An integrated pathway between primary care and secondary care may provide the optional solution for ADHD assessment. Hassink-Franke et al. [52] in their study of Dutch PCPs highlighted that greater support and more constructive long-term relationships with secondary care enhanced PCP’s confidence about ADHD. Greater support for Dutch PCPs also allowed families of children with ADHD to received care from PCPs with whom they had a long-lasting relationship and allowed care to be provided in a more informal primary care context rather than more formal secondary care.

## Discussion

This review concurs with findings from previous reviews [6.21] on the subject but by adding a larger body of literature, two new themes of internal and resource constraints and multidisciplinary approach were explored. It has found that a considerable number of barriers and facilitators such as lack of education, time and resource constraints, misconceptions and integrated pathways prevent PCPs from effectively supporting ADHD patients. By identifying these factors affecting access to care, this review establishes multiple areas of needs, enabling recommendations to facilitate PCPs’ ability in identifying and managing ADHD.

The need for education was the most highly endorsed factor overall, with PCPs reporting a general lack of education on ADHD. This need for education was observed on a worldwide scale; this factor was discussed in over 75% of our studies, in 12 different countries, suggesting that lack of education and inadequate education was the main barrier to understanding of ADHD in primary care. While this review reported both barriers and facilitators, barriers were mostly identified with very few facilitators. Overall, PCPs held a keen interest in gaining knowledge on ADHD, educational programs helped increase this knowledge and, over time, improved knowledge has been noticed. Studies investigating the presence of shared cared and integrated pathways reflect it to be the optimal solution. In conclusion, the main facilitator encompassing all themes in this review highlights the importance of providing any form of resources that would help PCPs facilitate access to care for individuals with ADHD.

However, resource constraints overall were an important barrier. While this factor was discussed as a separate theme, it also encompasses several other themes. Indeed, time and financial constraints affect the opportunities for PCPs to seek extra training and education but also affect the communication with other professionals such as secondary care workers, teachers and parents. This highlights further the difficulties faced daily by PCPs in recognising and managing patients with ADHD.

### Strength and limitations

This review included different methodologies, qualitative, mixed methods and quantitative studies. Following the methods presented in our analyses, studies were considered separately (according to their methodology) at the analysis stage. We expected different methodologies to highlight different findings with one adding extra information to the other; however, for the majority of results, this was not the case. The different methodologies highlighted similar factors in understanding access to care for people with ADHD.

This review included a broad sample of studies from a worldwide perspective. Similar barriers were identified internationally, highlighting that these factors may not be culture dependent and appear to be widely generalisable. However, the majority of the studies were based in developed, western countries and more research in this area from developing countries in Asia and South America may allow subtler differences to emerge.

In many countries, pathways to care for adults and children are very distinct; therefore, divergent findings within adult and child studies might have been expected. However, no distinction was observed, with similar factors affecting both children and adults alike, determining that the barriers discussed in this review are relevant to different age groups and systems to care.

This review focused primarily on PCPs’ understanding and knowledge of ADHD, by including studies principally focusing on PCPs. A small but significant number of studies also included views from other parties such as parents and other professionals. It was interesting to notice that their views were in agreement with the findings generally observed and were not conflicting, adding validity to our overall observations.

By including different methodologies from multiple languages and following a strict systematic approach with clear transparency of the review process (including quality assessment, multiple reviewers and thorough data extraction method) this review included all relevant published studies on the subject and minimised the risk of biases.

However, a few limitations can be observed in this review. There was considerable variability in the quality of the included studies. Studies also varied considerably in the extent to which they contributed to the review, with some studies bearing more weight on our observations.

Barriers and facilitators were initially defined in order for this review to identify them as accurately as possible. However, most studies did not explicitly mention the terms ‘barriers’ and ‘facilitators’ and, therefore, these concepts were subject to our interpretations.

Only a small proportion of studies included in this review were published recently (11/48 studies since 2010). Thus, it is possible that while these findings were more relevant a decade or so ago, they might not be as significant if focused only on recent studies. While unlikely, possible reasons for fewer recent studies in this area might be that these issues are no longer as salient or that fewer studies are required as existing findings are still felt to be relevant. Further research is required in this important area.

Studies adopted different methodological approaches, including six qualitative and two mixed-method studies. While direct comparison between different methodological approaches was limited, most of this review’s findings were supported by both quantitative and qualitative studies with the exception of the role of the media which was only highlighted by qualitative studies.

It is important to note that the sample selected by these studies is selective. It has been observed that some PCPs do not believe in ADHD [74]. Therefore, it could be assumed that participating PCPs would likely have some openness or strong views about ADHD to take part. PCPs having strong beliefs about the existence (or not) of ADHD might not have been willing to partake in these studies and, therefore, their representation will be lacking from our findings. Finally, as this was a systematic review rather than a meta-analysis it was not possible to explore publication bias and its impact on study conclusions and our review.

### Implication for practice

The potential barriers faced with knowledge of ADHD in primary care may lead to underdiagnosis or misdiagnosis, delays in being referred and lack of access to the right support [15]. Highlighting knowledge gaps can inform the development of future research, targeted interventions or psychoeducation programs for established PCPs as well as professionals in training. Increasing accurate knowledge of ADHD within this chosen population could improve recognition rates, benefiting patients and healthcare workers alike. Improvement in diagnosis could subsequently follow, either by more timely referral to secondary care services which are responsible for diagnosis (for instance, in UK population) or by quicker diagnosis in settings where PCPs are able to make a diagnosis (for instance, in US population). Better training of PCPs on ADHD is, therefore, necessary but to facilitate this, dedicated time and resources towards education needs to be put in place by service provider and local authorities. While the development of educational programs for PCPs seems to be the most characterised need, this issue requires further exploration and investigation as only two studies that investigated the benefits of an interventional program on PCPs [52, 56] were identified in this review, both with limited generalisability.

### Implications for research

Although the need for the development of educational programs is strongly present, before instituting such programs in primary care settings, research on relevant and appropriate methods needs to be conducted. Developing the right intervention is essential as PCPs have very limited time and a lengthy full-day workshop, for instance, would not be easily accessible or provided for this population. Future research will also need to address more specifically the factors of resource constraints, misconceptions and multidisciplinary approaches, to overcome more specific challenges. These findings can then be used to develop more targeted strategies in enhancing access to care for ADHD.

While most studies in this review were quantitative, we believe mixed methods studies would be more beneficial in investigating these factors. Quantifying the effect of such factors on access to care is important but gaining an insight into the experience and attitudes of PCPs adds valuable knowledge on their individual beliefs, awareness and experience that is difficult to access through quantitative methods. In the context of this review, for instance, the link made between misconceptions and the role of the media and label was only made through the use of qualitative enquiries, quantitative methods might not have allowed this theme to emerge.

It is important to note that while this review focuses on primary care, our findings and previous studies [6] suggest that training teachers and parents could also be strongly beneficial in the process of continuing access to care for ADHD.

## Electronic supplementary material

Below is the link to the electronic supplementary material.

Supplementary material 1 (DOCX 14 kb)

Supplementary material 2 (DOCX 14 kb)
