# Supplementary material for: Barriers and facilitators to understanding of ADHD in primary care: a mixed-method systematic review
Source: Eur Child Adolesc Psychiatry. 2018 Dec 14;28(8):1037–64. doi: 10.1007/s00787-018-1256-3 (PMC6675769; doi:10.1007/s00787-018-1256-3)
Supplement: Supplementary file 1 — Supplementary material 1 (DOCX 14 kb) [file 787_2018_1256_MOESM1_ESM.docx]

Supplementary table S1

Record of reasons for excluding studies after full text assessment

1. Aksoy, U. M., et al. (2015). Not on primary care

2. Ayyash, H., et al. (2013). Not peer reviewed

3. Beirne, M., et al. (2013). Not primary care

4. Bennett, F. C. and R. Sherman (1983). No mention of attitudes

5. Blew, H. and G. Kenny (2006). Not attitudes

6. Bunik, M., et al. (2013). Not attitudes

7. Bussing, R., et al. (2003). Not attitudes, not primary care

8. Bussing, R., et al. (2012). Not primary care

9. Cardo, E., et al. (2017). Not primary care

10. Dennis et al. (2008) Not just primary care

11. Efron, D., et al. (2016). Not primary care

12. Epstein, J. N., et al. (2007). Not attitudes

13. Epstein, J. N., et al. (2008). Not attitudes

14. Epstein, J. N., et al. (2010). Not attitudes

15. Epstein, J. N., et al. (2010). Not attitudes

16. Epstein, J. N., et al. (2013). Not attitudes

17. Fitzgerald, M. and F. McNicholas (2014). Not just primary care

18. Foy, J. M. and M. F. Earls (2005). Case study

19. Frigerio, A., et al. (2013). Not primary care

20. Gardner, W., et al. (2004). Not attitude

21. Glod, C. A. (2001). Full text not available

22. Groen, W., et al. (2006). Case study

23. Gross, B. (2005). Opinion piece

24. Guevara, J. P., et al. (2008). No mention of attitudes

25. Hall, C. L., et al. (2013). Not primary care

26. Hays, R. B. (1999). Opinion piece

27. Hazelwood, E., et al. (2002). Not primary care

28. Healy, D., et al. (2013). Not attitudes

29. Hill, P. and M. Cameron (1999). Not primary care

30. Hinshaw, S. P., et al. (2011). Not primary care

31. Hirfano?lu, T., et al. (2008). Not sure if primary care

32. Homer, C. J., et al. (2004). Not attitudes

33. Huss, M., et al. (2008). Not attitudes

34. Jensen, C. M. and H. C. Steinhausen (2015). Not attitudes

35. Khalil, M. S. and E. Jenahi (2015). Not primary care

36. Lazorick, S., et al. (2008). Not attitudes

37. Leitner, Y., et al. (2016). Not attitude

38. Lobar, S. L., et al. (1999). Not primary care

39. Luk, E. S. L. (2002). Opinion piece

40. Mann, E. M., et al. (1992). Not primary care

41. Marcer, H., et al. (2008). Not primary care

42. McCarthy, S., et al. (2013). Not attitudes

43. McClain, M. R., et al. (2014). Not ADHD specific

44. Montano, C. B. and J. Young (2012). Literature review

45. Morley, C. P. (2010). Opinion piece

46. Moser, S. E. and K. J. Kallail (1995). Not attitudes

47. Mueller, A. K., et al. (2012). Literature review

48. Noury, J. L., et al. (2010). Not primary care

49. O'Keeffe, N. and F. McNicholas (2011). Not primary care

50. Olfson, M. (2010). Not peer reviewed- editorial

51. Oshodi, Y. O., et al. (2012). Not primary care

52. Patel, A., et al. (2016). Not peer reviewed

53. Rafalovich, A. (2005). Not just primary care

54. Ramsay, J. R. (2014). Opinion piece

55. Reale, L., et al. (2015). Not primary care

56. Salmon, G. and A. Kemp (2002). Not primary care

57. Sax, L. and K. J. Kautz (2003). Not attitude

58. Senecky, Y., et al. (2007). Not ADHD specific

59. Sheldrick, R. C., et al. (2015). Not just primary care

60. Sundet, R. (2011). Not ADHD specific

61. Swift, K. D., et al. (2014). Literature review

62. Thapar, A. and A. Thapar (2002). Opinion piece

63. The, L. (2007). Not attitude

64. Toomey, S. L., et al. (2008). Not attitude

65. Toomey, S. L., et al. (2011). Not attitude

66. Venter, A., et al. (2004). Not primary care

67. Vierhile, A., et al. (2009). Opinion piece

68. Voigt, R. G. and P. J. Accardo (2016). Opinion piece

69. Waite, R. (2007). Literature review

70. Walton, J. et al. (2014). Not attitudes

71. Wetzel, M. W. (2009). Not primary care

72. Whitely, M. (2013). Opinion piece

73. Wolraich, M. L. (1999). Review

74. Wolraich, M. L. (2002). Opinion piece

75. Wolraich, M. L., et al. (2005). Not attitude

76. Young, S., et al. (2011). Review

77. Zima, B. T., et al. (2013). Not just primary care
